# Hairy-root organ cultures for the production of human acetylcholinesterase

**DOI:** 10.1186/1472-6750-8-95

**Published:** 2008-12-23

**Authors:** Ryan R Woods, Brian C Geyer, Tsafrir S Mor

**Affiliations:** 1 School of Life Sciences and The Biodesign Institute, P.O. Box 874501, Arizona State University, Tempe, AZ 85287-4501, USA

## Abstract

**Background:**

Human cholinesterases can be used as a bioscavenger of organophosphate toxins used as pesticides and chemical warfare nerve agents. The practicality of this approach depends on the availability of the human enzymes, but because of inherent supply and regulatory constraints, a suitable production system is yet to be identified.

**Results:**

As a promising alternative, we report the creation of "hairy root" organ cultures derived via *Agrobacterium rhizogenes*-mediated transformation from human acetylcholinesterase-expressing transgenic *Nicotiana benthamiana *plants. Acetylcholinesterase-expressing hairy root cultures had a slower growth rate, reached to the stationary phase faster and grew to lower maximal densities as compared to wild type control cultures. Acetylcholinesterase accumulated to levels of up to 3.3% of total soluble protein, ~3 fold higher than the expression level observed in the parental plant. The enzyme was purified to electrophoretic homogeneity. Enzymatic properties were nearly identical to those of the transgenic plant-derived enzyme as well as to those of mammalian cell culture derived enzyme. Pharmacokinetic properties of the hairy-root culture derived enzyme demonstrated a biphasic clearing profile. We demonstrate that master banking of plant material is possible by storage at 4°C for up to 5 months.

**Conclusion:**

Our results support the feasibility of using plant organ cultures as a successful alternative to traditional transgenic plant and mammalian cell culture technologies.

## Background

Bioscavenging of organophosphate (OP) by human cholinesterases (ChEs) is emerging as a promising medical intervention for prophylaxis and post-exposure treatment against chemical warfare nerve agents and pesticides, meeting considerable success in pre-clinical studies [1,2]. ChEs are very efficient in sequestering OPs that become esterified to a serine residue at the active site. This covalent bond is very stable and in the case of certain OPs is further stabilized by subsequent "aging" reactions. With the phosphorylated enzymes having negligible reactivation rates, ChEs are effectively "single-use molecular sponges" requiring the application of stoichiometric rather than catalytic doses for effectiveness. Thus a production system capable of supplying the forecasted demand for large amounts of active ChEs is needed.

Several strategies for production of ChEs were evaluated. Of the two ChEs in humans, only the serum enzyme butyrylcholinesterase (BChE) can be obtained from natural sources, and large-scale purification efforts from outdated blood-banked human plasma were demonstrated [2-4]. Expression in recombinant systems is the only way for producing the physiological target of OPs, acetylcholinesterase (AChE), which is abundant in muscle and nerve tissues but is normally absent from serum. Several mammalian-based recombinant production systems were described including engineered cell cultures [e.g. [5-7]] and the milk of transgenic goats [8]. As an alternative to these systems that are confronted with being supply-restricted, of limited scalability, high cost and risk of human pathogen contamination, we have introduced plants as a production system for human AChE [9]. We carefully optimized the expression constructs [10], and purification protocols [11] and demonstrated that plant derived AChE-R_ER _from *Nicotiana benthamiana*, which retains all of the catalytic properties of a mammalian-derived enzyme and furthermore that plant-derived AChE-R_ER _is capable of completely ameliorating all of the gross clinical symptoms and some of the long-term molecular consequences implicated in OP poisoning [12].

Despite their promise, there are currently some concerns among regulatory agencies and the public at large, regarding the use of transgenic plants (grown in open fields or in greenhouses) for the production of protein pharmaceuticals [13]. Among the raised issues is that of environmental containment (both in term of transgene escape and inadvertent contamination of non-transgenic plant material). A further perceived difficulty is the regulatory uncertainty whether the lack of tightly controlled growth conditions typical of plant cultivation can satisfy the strict requirements of good manufacturing practice. In this context plant cell or organ cultures grown in bioreactors may prove more adept at clearing the regulatory hurdles associated with plant-based heterologous production systems while maintaining their most important advantages – inexpensive medium consisting of salts and sugar and devoid of mammalian proteins, growth factors and hormones; equivalent purification costs; and unmatched biosafety bearing minimal risk of human pathogens and prions [13].

While several plant cell lines are available for use, more organized organ culture, such as "hairy root" cultures may present additional benefits e.g. genetic and biochemical stability and faster growth rates resulting in larger mass/medium ratios [14,15]. Hairy root cultures are obtained by *Agrobacterium rhizogenes *mediated transformation of plant tissue (explants). *A. rhizogenes *is a common phytopathogenic and naturally-transforming soil bacterium (for a review see Guillon et al. [16]. It induces neoplastic growth and differentiation of infected plant tissue to form "hairy roots" by activation of genes on a DNA fragment (T-DNA) that is transferred from the bacterial Ri plasmid and integrated into the plant nuclear genome. The transformed plant tissue quickly grows into a highly branched mass in a medium consisting of simple salts and sucrose and useful compounds such as secondary metabolites or recombinant proteins can be recovered from the medium or extracted from the plant tissue [16].

Here we demonstrate the feasibility of producing human AChE-R_ER _in hairy root cultures derived from transgenic *N. benthamiana *plants expressing the protein via *A. rhizogenes *mediated transformation. Hairy root lines were screened for level of expression of AChE-R_ER _and the protein was subsequently purified and its biochemical properties studied and its circulation half-life determined.

## Methods

### Cloning, Tissue Culture and Initial Screening

**Figure 1:**
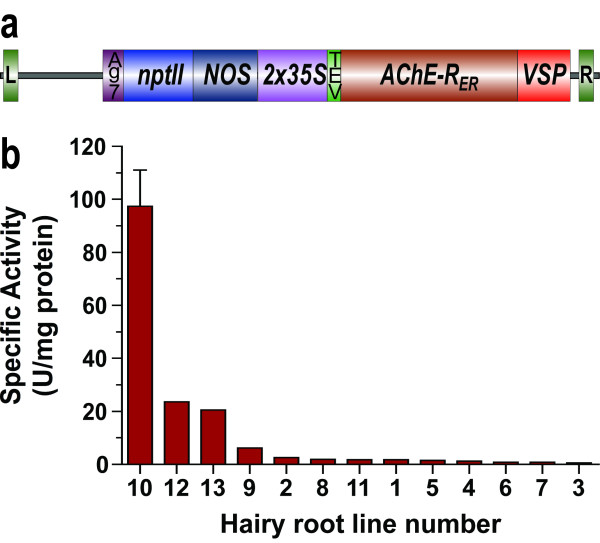
**a: Expression cassette driving the production of human AChE-R_ER _in transgenic plants and hairy-root cultures**. L, left border; R, right border; Ag7, the nopaline synthase gene's 3' UTR; nptII, the kanamycin resistance gene; NOS, the nopaline synthase gene's promoter; 2 × 35S, cauliflower mosaic virus 35S promoter with duplicated enhancer; TEV, the translation enhancer region of the tobacco etch virus; AChE-R_ER_, codon-optimized coding region for human AChE-R containing the C-terminal SEKDEL ER retention signal; VSP, the 3' UTR of the soybean vegetative storage protein. b: Levels of AChE-RER expression in several independent hairy-root clones.

A codon-optimized cDNA encoding human AChE-R with a C-terminal SEKDEL (Fig. 1) was as previously synthesized by de novo assembly and stable *N. benthamiana *lines were established [12]. Explants derived from these parental plant lines, along side untransformed wild type (WT) plants, were co-cultivated with *Agrobacterium rhizogenes *(R 1000) for 48 hr in the dark at room temperature on plates containing MS medium containing MS basal salt mixture with vitamins (Phytotechlabs, 4.3 g/L), 3% sucrose (Sigma) and 0.2% phytagel (Sigma). Explants were then moved to fresh MS medium supplemented with 50 mg/L kanamycin (Phytotechlabs) and 300 mg/L Timentin (Phytotechlabs). Hairy roots thus obtained were grown on solid MS medium or and in 1 L flasks containing 500 mL liquid culture shaken at 100 RPM.

For initial screening, root samples (100 mg) were lysed in 3 vol of extraction buffer (50 mM Tris, pH 8, 1 M NaCl, 1% Triton X-100) using a FastPrep machine (Qbiogene). Lysates were then clarified by centrifugation at 14,000 × g for 10 min at 4°C. Supernatants were removed and enzymatic activity of AChE-R was measured (see below).

### Determination of Growth Kinetics and 4°C storage protocol

Liquid culture growth rate and maximum culture density were determined for hairy roots lines expressing AChE-R_ER _as well as WT roots. MS liquid medium (500 mL) was spiked with 1.5 g hairy root tissue that had been grown on solid media for 2 weeks. Roots were removed from liquid culture and dried briefly on paper towels at the corresponding time points. For AChE purification experiments, hairy root cultures were typically harvested between 4–6 weeks following culture initiation, ensuring maximum culture density. For 4°C storage experiments, roots were grown on 1.5% agar (Sigma) slants containing MS salts and 3% sucrose and allowed to grow at room temperature for 3 weeks in the dark. Slants were then moved to 4°C. At the indicated times, slants were removed and roots were sterilely transferred to 50 mL of liquid MS medium and cultured on a shaker at RT for 3 weeks. Roots were then removed, patted-dry, inspected and weighed.

### Purification of Human Acetylcholinesterase

All purification steps were preformed at 4°C. After 4–6 weeks of growth remaining media was removed from the growth flask and roots were washed once with 500 mL extraction buffer (10% sucrose, 5 mM MgCl_2_, 15 mM Na_2_S_2_O_5 _in PBS, pH 8.0). Root samples were disrupted in a commercial blender with 2 volumes of fresh extraction buffer and subjected to centrifugation at 20,000 × g for 20 minutes to pellet cellular debris. The supernatant was filtered first with Miracloth (Calbiochem) followed with a Grade 50 filter paper (2.7 μm, Whatman). The clarified supernatant was then diluted with PBS to two times initial volume to reduce the metabisulfite concentration and processed by affinity chromatography using 8 mL procainamide-agarose (Sigma) in a 2.5 cm I.D. Econo-column (Bio-Rad). The column was washed with 80 mL PBS and the bound enzyme eluted with 60 mL elution buffer (0.2 M acetylcholine chloride in PBS) into 2 mL fractions. Fractions displaying AChE activity (2–18) were pooled, ammonium sulfate was added to 55%, and the suspension incubated for 1 hr. The protein pellet obtained by centrifugation (20,000 × g, 15 min) was re-suspended in 20 mL buffer 1 (20 mM Na_2_PO_4_/NaHPO_4_, 20 mM NaCl, pH 7.4), dialyzed extensively against buffer 1 for 12 hr in a 50 kDa MWCO cellulose dialysis tubing (Spectrum), and concentrated in a Macrosep 10 kDa MWCO concentrator (Pall) to a final volume of 3 mL. Sodium azide (0.02%) was added and solution was stored at 4°C until further use.

### Biochemical characterization of AChE-R

Enzymatic activity of AChE-R was measured using acetylthiocholine iodine (ATChI, Sigma) as the substrate in a SpectraMax 340PC spectrophotometer (Molecular Devices) by the method of Ellman [17] as previously described [9]. The specific activity of pure preparations of plant-derived AChE (~3000 U/mg protein [12]) was used to convert activity protein equivalent. Total protein levels were derived using the Bio-Rad Protein Assay Reagent (Bio-Rad) with BSA as the standard. The Michaelis constant (K_M_) was determined by measuring and plotting AChE activity as a function of ATChI concentration and non-linear regression analysis (Prism software, GraphPad). Inhibition curves were generated by plotting residual AChE activity (measured in the presence of 1 mM ATChI) as a function of inhibitor's concentration using the following acetylcholinesterase inhibitors: neostigmine bromide (Sigma), 1,5-bis(allyldimethylammoniumphenyl)pentan-3-one dibromide (BW, Sigma), diethyl p-nitrophenyl phosphate (Paraoxon, Sigma) and the butyrylcholinesterase specific irreversible inhibitor, tetra-isopropyl pyrophosphoramide (ISO-OMPA, Sigma).

Protein samples were resolved by SDS-PAGE and visualized using a Silver Snap II kit (Pierce) according to manufacturer's instructions.

### Pharmacokinetics

Groups of five 6–8 week old male FVB/N mice were injected with 30 U of hairy root-derived AChE-R_ER _in PBS (100 μL) or 100 μL PBS as vehicle control. Blood samples (25 μL) were drawn by tail vein knick. Serum was separated from clotted blood by centrifugation (6,000 × g, 30 min, 4°C). Serum samples were assayed for AChE activity in the presence of 50 μM of the butyrylcholinesterase-specific inhibitor Iso-OMPA. Data derived from AChE-injected mice was normalized to data derived from vehicle-treated mice.

## Results and Discussion

### Creation of hairy root cultures

Previously we described the creation of transgenic *N. benthamiana *plants expressing the "readthrough" isoform of human AChE (AChE-R) [11,12]. This isoform is the monomeric and soluble product of one of the mRNA splice variants of the single human *ACHE *gene, which is up-regulated during exposure to anticholinesterase agents [18]. The human enzyme was engineered to contain the endoplasmic reticulum (ER) retention signal KDEL (Fig. 1a). This modification, as well as codon optimization of the gene, enabled the recombinant protein to accumulate to high levels in leaves [10,12]. We selected one of the highest expresser line, 2D, harboring (at least) four copies of the transgene and expressing AChE-R_ER _at 0.3%–1% of total soluble protein (TSP) as the parental source of explants for the generation of hairy root cultures [11,12].

Thirteen independent hairy root lines were generated by *A. rhizogenes *infection and screened for the presence of the transgene and for levels of recombinant protein, assayed by its enzymatic activity (Fig. 1b). These kanamycin-resistant clones displayed a wide distribution of recombinant protein accumulation, with the highest clone accumulating the transgenic product at 3.3% TSP while the lowest confirmed positive clones expressed at levels that were at least 100 fold lower. We observed a similarly wide distribution of recombinant protein accumulation levels in hairy-root clones derived from a single parental plant with the synaptic AChE isoform (data not shown).

The differences in the apparent expression levels of AChE-R_ER _between the hairy root cultures and the parental plants can be explained by several, non-mutually exclusive explanations. For example it is reasonable to expect differences in the size of total soluble protein fraction in roots vs. leaves. It can also reflect a less restrained transgene expression under the pampered culture conditions as opposed to expression levels that can be expected of potted plants in the greenhouse. Regardless of the explanation, the higher % TSP observed in hairy roots presents itself as a bonus when purification is concerned.

### Growth kinetics of AChE-R_ER _hairy root cultures

**Figure 2:**
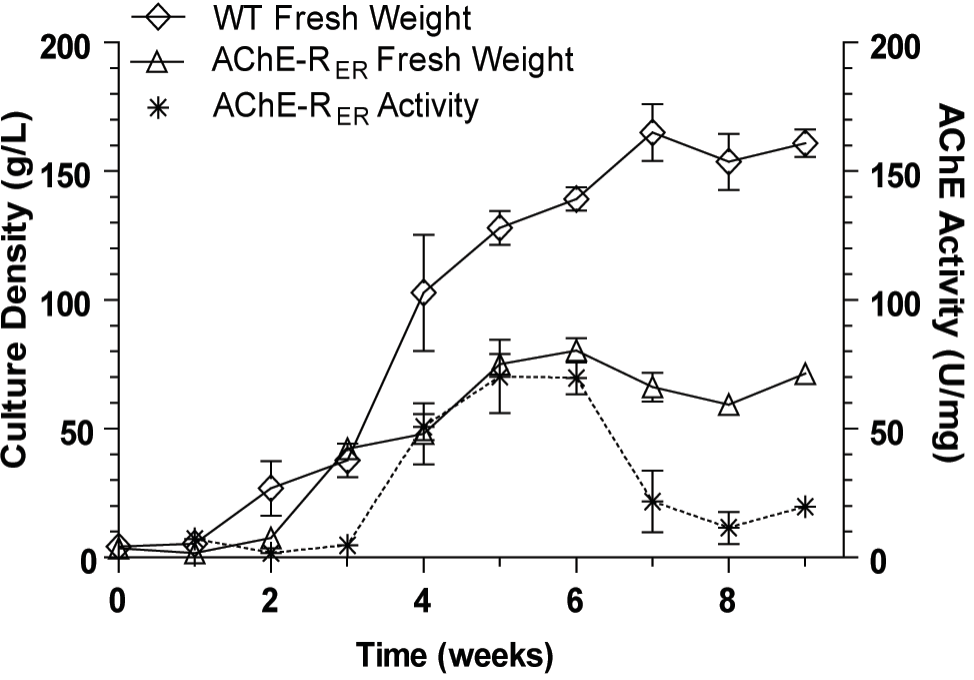
**Growth profile of hairy root cultures derived from clone 10 (Fig. 1) as compared to hairy-root cultures derived from untransformed *N. benthamiana *plants**. Shown are fresh weight gains and accumulation of AChE activity in tissue. Plotted values indicate the mean ± S.E.M of three independent repeats.

Following selection and initial screening, the clone with the highest AChE-R_ER _accumulation (clone 10) was selected for further studies and was cultured in liquid medium. Under identical growth conditions, transgenic hairy root cultures were slower to grow and reached stationary phase earlier and at lower densities than WT cultures (Fig. 2b). Thus, transgenic cultures reached peak density (76 g/L, Fig. 2) already at 5 weeks, when growth rate tempered while the WT cultured continued to grow at a vigorous pace up to 7 weeks after inoculation and achieved roughly twice that density (161 g/L, Fig. 2). Such differences between the growth rates of the transgenic vs. WT cultures were not evident when hairy roots were grown on solid medium. This may indicate that the rate-limiting factor is aeration, although any other number of factors may contribute as well. Further optimization of growth conditions may increase the growth rates of the cultures and alleviate the observed limitations. Regarding production of recombinant AChE-R_ER_, the culture productivity also peaked at week 5, when AChE specific activity in the crude extract was 70 U/mg (or about 2.3% TSP). Although the stationary phase culture remained viable, accumulation levels of the recombinant protein dropped sharply, possibly reflecting decreased stability of the enzyme due to senescence-induced proteases potentially coupled to decreased translation.

WT hairy roots remained viable when stored at 4°C on solid medium (agar slants) and could be used to inoculate new suspension cultures for at least 6 months. However, and correlating well to its diminished growth characteristics, AChE-expressing hairy roots remained viable for only 15–20 weeks. Methods for longer-term clone banking are still being pursued.

### Purification of AChE-R_ER _from hairy root cultures

**Figure 3:**
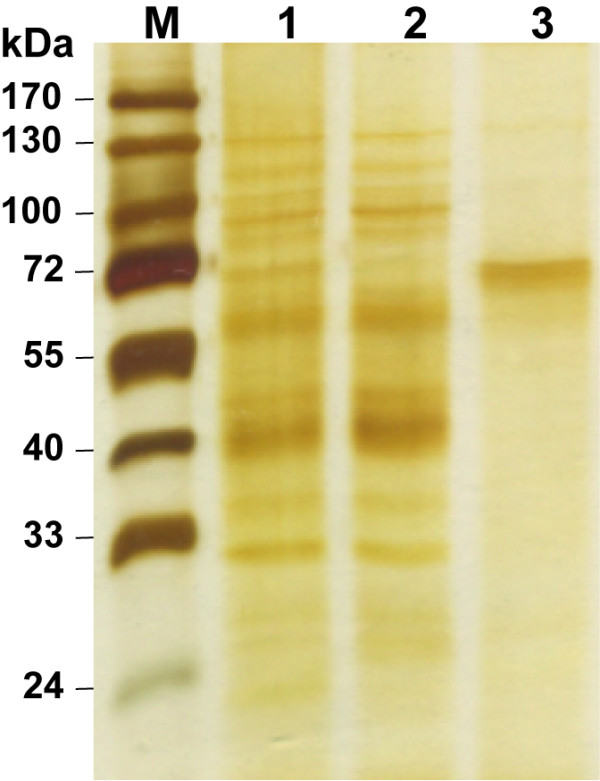
**Protein samples from successive purification steps were subjected to SDS-PAGE and visualized by silver staining as follows: lane 1 – crude extract; lane 2 – procainamide affinity chromatography flow-through; lane 3 – eluate after extensive dialysis; M – molecular weight standards**.

**Figure 4:**
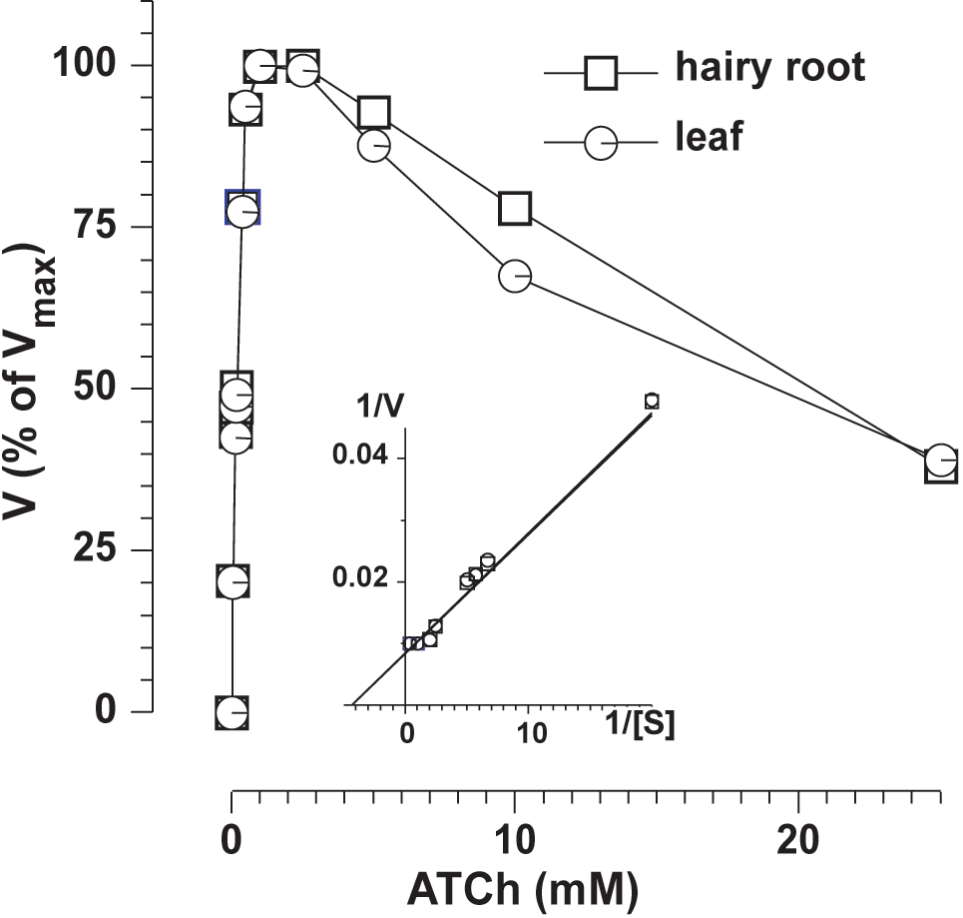
**Transgenic plant-produced (leaf) and hairy-root derived (hairy-root) AChE-R_ER _enzymes have identical K_M _values (Lineweaver-Burke analysis, insert) and substrate inhibition profiles**.

Following growth in liquid culture for 4–6 weeks, the recombinant protein was purified by affinity chromatography and concentrated by ammonium sulfate precipitation. The enhanced accumulation of AChE-R_ER _in hairy root cultures compared with the parental plants made additional purification steps [e.g. anion-exchange chromatography, see [11,12]] unnecessary to achieve comparable purity and yield to our previously published results [Fig. 3, Table 1. For comparison see [11,12]]. This isolation procedure resulted in an electrophoretically pure AChE-R_ER _with an overall 50-fold purification (Fig. 3, Table 1). Importantly, neither the choice of the hairy root system, nor the abbreviated purification protocol had any effect on the catalytic characteristics of the recombinant molecule. The hairy root derived enzyme's affinity to the substrate, approximated by the Michaelis constant (*K*_M_), was identical to that of AChE-R_ER _derived from the parental plant (0.23 ± 0.04 mM, mean ± SEM), and is also very similar to the native enzyme's value [19,20] (Fig. 4). Similarly, binding of substrate to the peripheral anionic site of AChE, responsible for its characteristic allosteric inhibition, was apparent at concentrations higher than 2.5 mM regardless of the source of the enzyme.

**Table 1:** Hairy-root derived recombinant AChE-R_ER _purification.

	**Total Protein**	**Total Activity**	**Specific Activity**	**Yield**	**Purification**
	***(mg)***	***(units)***	***(units/mg protein)***	***(%)***	***(fold)***
Crude Extract	40,000	2,300	57	100	1
Affinity Chromatography	60	171	2,800	7	49

**Figure 5:**
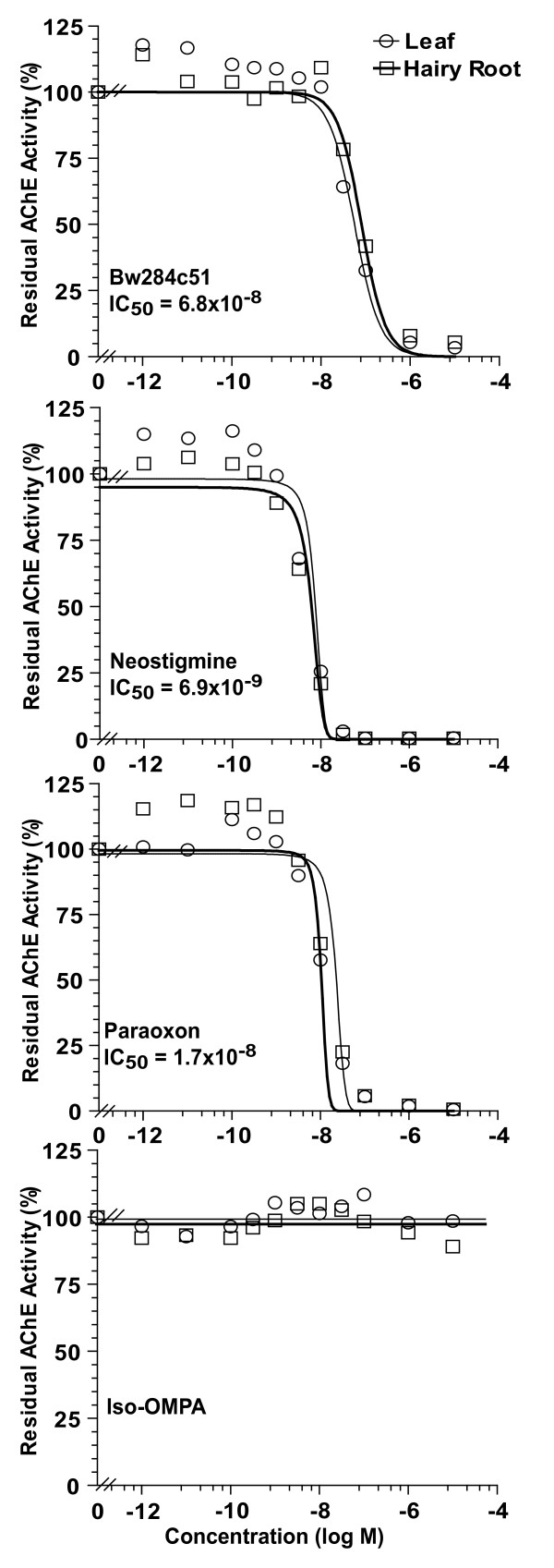
**Recombinant AChE-R derived from either leaves of transgenic plants or hairy-root cultures have identical sensitivities to various cholinesterase inhibitors**. Residual activities were measured following incubation with the indicated concentration of the indicated inhibitors.

As the goal of cholinesterase therapy is to provide a broad-spectrum bioscavenger of anti-cholinesterase toxins, binding of inhibitors to the enzyme was monitored by obtaining inhibition profiles for several cholinesterase inhibitors. The hairy-root derived enzyme showed nearly identical IC50 values with representatives of several classes of inhibitors including paraoxon, the active OP metabolite of the insecticide parathion), the AChE-specific bis-quaternary inhibitor BW 284-c51, and neostigmine bromide, a carbamate (Fig. 5a–c). As expected, Tetra(monoisopropyl)pyrophosphortetramide (Iso-OMPA), a butyrylcholinesterase-specific OP, had no effect on the plant-derived AChE (Fig. 5d).

The purification results presented here, demonstrate the potential for further improvements in increasing the yield. A relative simple modification may be, for example, using a variant of the protein devoid of the ER retention signal, which should allow the protein to be secreted to the apoplast. It is yet to be seen if such approach would allow the ~70 kDa protein to be released into the medium (as opposed to being trapped within the plant cell wall and subsequent uptake and degradation) as the existing literature is ambivalent about the issue [21-23].

### Pharmacokinetics of Hairy Root-Derived AChE-R_ER_

**Figure 6:**
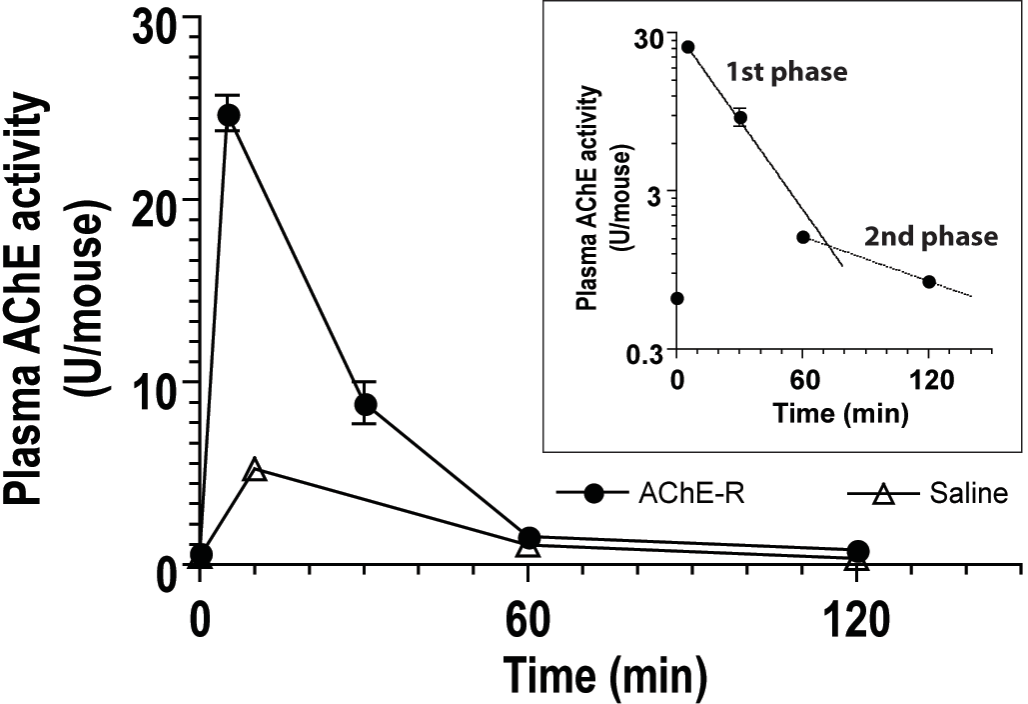
**Pharmacokinetics of hairy-root derived AChE-R_ER_**. Groups of 5 mice were injected with either 100 U recombinant AChE-R_ER _or an equivalent volume of PBS. Plasma samples were collected and assayed for AChE activity in the presence of Iso-OMPA, a selective BChE inhibitor. In the insert, results are fitted to two exponential elimination equations: 1^st ^phase: y = 30.8e^-0.043t ^and 2^nd ^phase: y = 2.97e^-0.011t^.

The levels of AChE activity in serum are typically very low but increase following various stressful insults as is demonstrated here with mice injected with vehicle (saline). Following the injection, the mice experienced a ten-fold increase in their serum AChE activity, which declined rapidly (Fig. 6). To determine the rate of circulatory clearance of hairy root-derived AChE-R_ER_, we injected (i.v.) mice with 25 U of the recombinant enzyme and tested plasma samples for residual AChE activity in the presence of Iso-OMPA (to inhibit serum BChE activity, Fig. 6). The hairy root-derived human enzyme was cleared rapidly with a two-phase exponential decay kinetics (Fig. 6 insert, K_1 _= 0.0433 ± 0.0037 min^-1 ^and K_2 _= 0.0109 ± 0.0022 min^-1 ^corresponding to half-life values of 16 min and 64 min, respectively). The kinetics of serum clearance of the hairy root-derived AChE-R is not appreciably different than that of the endogenous enzyme, probably reflecting the existing mechanisms to retain the normally low levels of activity in the serum [24]. Stability can be increased by decorating the enzyme with polyethylene glycol on surface exposed lysine residues [24].

## Conclusion

Previously, we reported the creation of transgenic plants that accumulate recombinant human AChE-R_ER _to commercially viable levels [12]. Here we demonstrate that the enzyme can be efficiently produced in hairy root cultures derived from those transgenic plants, that it can be readily purified and that it is 'biosimilar', i.e. biochemically and functionally equivalent to its transgenic plant-derived counterpart with respect to substrate hydrolysis, OP binding and pharmacokinetics [12]. It is anticipated, but yet to be demonstrated, that the hairy root enzyme can provide similar protection to OP challenged enzymes. Thus organ cultures can provide both the high level of expression achieved with transgenic plants, with the additional containment and uniformity coming from contained clonal propagation in well-defined culture medium and conditions.

## Authors' contributions

RRW conducted the *A. rhizogenes *transformation experiments, obtained growth parameter analysis, carried out many of the biochemical assays and drafted the manuscript. BCG created the expression construct and created the transgenic plants from which the hairy-root cultures were derived and participated in all of the above activities. TSM conceived of the study and participated in its design and coordination as well as wrote the final version of the paper in its originally submitted and revised forms. All authors read and approved the final manuscript.
